# Strigolactones Might Regulate Ovule Development after Fertilization in *Xanthoceras sorbifolium*

**DOI:** 10.3390/ijms25063276

**Published:** 2024-03-14

**Authors:** Qingyuan Zhou, Linyi Zhou, Qing Cai

**Affiliations:** 1 Institute of Botany, Chinese Academy of Sciences, Xiangshan, Beijing 100093, China; caiqing@ibcas.ac.cn; 2 College of Life Sciences, Beijing Normal University, Beijing 100875, China; linyizho@163.com

**Keywords:** fertilized ovules, invertase, strigolactone signals, sugar, *Xanthoceras sorbifolium*

## Abstract

Strigolactones (SLs) were recently defined as a novel class of plant hormones that act as key regulators of diverse developmental processes and environmental responses. Much research has focused on SL biosynthesis and signaling in roots and shoots, but little is known about whether SLs are produced in early developing seeds and about their roles in ovule development after fertilization. This study revealed that the fertilized ovules and early developing pericarp in *Xanthoceras sorbifolium* produced minute amounts of two strigolactones: 5-deoxystrigol and strigol. Their content decreased in the plants with the addition of exogenous phosphate (Pi) compared to those without the Pi treatment. The exogenous application of an SL analog (GR24) and a specific inhibitor of SL biosynthesis (TIS108) affected early seed development and fruit set. In the *Xanthoceras* genome, we identified 69 potential homologs of genes involved in SL biological synthesis and signaling. Using RNA-seq to characterize the expression of these genes in the fertilized ovules, 37 genes were found to express differently in the fertilized ovules that were aborting compared to the normally developing ovules. A transcriptome analysis also revealed that in normally developing ovules after fertilization, 12 potential invertase genes were actively expressed. Hexoses (glucose and fructose) accumulated at high concentrations in normally developing ovules during syncytial endosperm development. In contrast, a low ratio of hexose and sucrose levels was detected in aborting ovules with a high strigolactone content. *XsD14* virus-induced gene silencing (VIGS) increased the hexose content in fertilized ovules and induced the proliferation of endosperm free nuclei, thereby promoting early seed development and fruit set. We propose that the crosstalk between sugar and strigolactone signals may be an important part of a system that accurately regulates the abortion of ovules after fertilization. This study is useful for understanding the mechanisms underlying ovule abortion, which will serve as a guide for genetic or chemical approaches to promote seed yield in *Xanthoceras*.

## 1. Introduction

Strigolactones (SLs) are a group of carotenoid-derived compounds that were recently defined as a novel class of plant hormones [1]. These signaling molecules were found to regulate diverse developmental processes [2,3,4,5,6] and environmental responses [7,8], such as inhibiting the outgrowth of axillary buds, regulating root development, accelerating leaf senescence, and mediating plants’ adaptation to nutrient deficiency. SLs were initially discovered because of their ability to stimulate the seed germination of root-parasitic plants such as *Striga lutea*. As a germination stimulant for *S. lutea*, strigol was the first SL that was isolated from the root exudate of cotton [9]. To date, more than 20 SLs have been extracted and identified in a variety of plant species [10]. Strigolactones are produced at high levels in the roots of various plant species under phosphate (Pi)-deficient conditions, stimulating changes in shoot and root architecture that enable them to adapt to environmental stress and improve their ability to acquire Pi [11,12].

SL-related mutants have been identified in several plant species, such as in Arabidopsis *more axillary growth* (*max*) mutants [13,14,15,16], *lateral branching oxidoreductase* (*lbo*) mutants [16], *suppressor of more axillary growth2-like* 6,7,8 (smaxl *6*,*7*,*8*) mutants [17], pea (*Pisum sativum*) *ramosus* (*rms*) mutants [18,19,20], petunia (*Petunia hybrida*) *decreased apical dominance* (*dad*) mutants [21,22], and rice (*Oryza sativa*) *dwarf* (*d*) or *high*-*tillering dwarf* (*htd*) mutants [23]. Based on studies with mutants, eight genes required for SL biosynthesis and signaling have been identified to date [24]. Among them, five have been shown to be involved in SL biosynthesis, including *D27*, *MAX3*/*D17*/*HTD1*/*RMS5*/*DAD3*, *MAX4*/*D10*/*RMS1*/*DAD1*, *MAX1,* and *LBO*, which encode β-carotene isomerase, carotenoid cleavage dioxygenase 7 (CCD7), CCD8, cytochrome P450 monooxygenase, 2-oxoglutarate, and Fe(II)-dependent dioxygenase (LBO), respectively. D27, CCD7, CCD8, and MAX1 have been identified as canonical SL biosynthetic enzymes [25]. The two-oxoglutarate-dependent dioxygenase LBO is considered to be a late-acting, noncanonical SL biosynthetic enzyme in Arabidopsis [17].

Three genes encoding an α/β fold hydrolase, an F-box leucine-rich protein, and a repressor protein known as D53 mediate the perception and signaling pathway of SLs. *D14* in rice [26], *AtD14* in Arabidopsis [27], *RMS3* in pea [28], and *DAD2* in petunia [29] encode an α/β-fold hydrolase that cleaves SLs and covalently binds to one of the cleavage products. This binding induces a conformational change in the receptor protein structure, leading to the activation of D14 [26,30]. The activated D14 interacts with the leucine-rich-repeat F-box protein encoded by *D3*/*MAX2*/*PhMAX2*/*RMS4* (in rice/Arabidopsis/petunia/pea, respectively) to form an Skp-Cullin-F-box (SCF) complex, SCF-D3-D14 [31]. This complex further activates the 26S proteasome and degrades transcription repressors encoded by *D53* in rice (*SMXL6*/*7*/*8* in Arabidopsis) in the presence of SL to trigger various SL-regulated plant responses [23].

Sugars not only provide a source of cellular energy and metabolic precursors for numerous biosynthetic pathways, but also function as signaling molecules to regulate gene expression in response to developmental and environmental cues [32,33]. A number of plant metabolic, physiological, and developmental processes are regulated in response to changing levels of soluble sugars [34]. Recent studies have provided evidence of interactions between sugar and the strigolactone response [35]. Increased sugar availability alleviates the inhibitory effect of strigolactone on shoot branching in pea, rose, chrysanthemum, and rice [36,37,38]. In many species studied, the rate of unloading and utilization of sucrose has been found to be correlated with the activity of invertases in sink organs [39]. Invertase activity is regulated by internal and environmental factors including plant hormones, carbohydrates, and mineral nutrition [40].

*Xanthoceras sorbifolium*, a tree species of the Sapindaceae family, is an emerging valuable oilseed crop in Northern China [41]. The plant has attracted considerable interest due to the high oil content (from 30% to 36% on a dry matter basis) in its seeds and its favorable oil qualities. The oil is considered an excellent source of unsaturated fatty acids because of its high oleic acid (ca. 28% of the total fatty acids) and linoleic acid (ca. 46%) contents [42]. *Xanthoceras* oil also contains high levels of vitamin E and nervonic acid, which have various health benefits. Despite its high-quality nutritional oil, its large-scale commercial cultivation has been hampered by low seed productivity. More than 95% of young fruits cannot reach maturity due to the abortion of ovules after fertilization within them. Although cytological observations of the development of fertilized ovules have been detailed in *X*. *sorbifolium* [41,43], much less attention has been paid to the regulatory mechanisms of ovule abortion in the plant. Strigolactones have been suggested to be important regulators of signaling and responses related to nutritional deficiencies [7]. This enticed us to examine whether SLs are produced in fertilized ovules and to assess whether SL signaling is correlated with Pi deficiency, invertase activity, and sugar availability in ovules after fertilization. Using multiple approaches, we attempted to determine whether SLs are involved in the abortion of fertilized ovules in *X*. *sorbifolium*. A better understanding of the mechanisms underlying ovule abortion will serve as a guide for genetic or chemical approaches to promote seed yield in *Xanthoceras*.

## 2. Results

### 2.1. The Presence of Strigolactones in Ovules after Fertilization and the Effect of Exogenous Pi Application on Their Content

**Figure 1:**
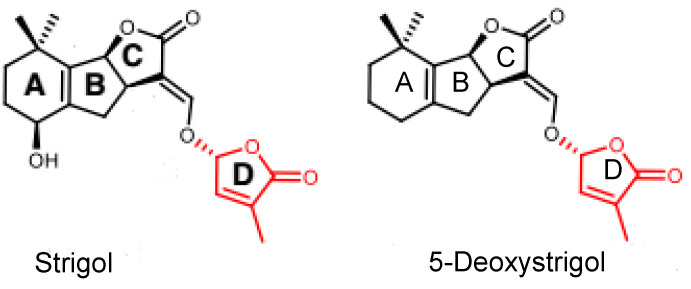
Chemical structures of two natural strigolactones (SLs). Both strigol and 5-deoxystrigol contain a methylbutenolide moiety (D-ring) connected to a variable tricyclic lactone (ABC-ring), which are termed canonical SLs.

**Figure 2:**
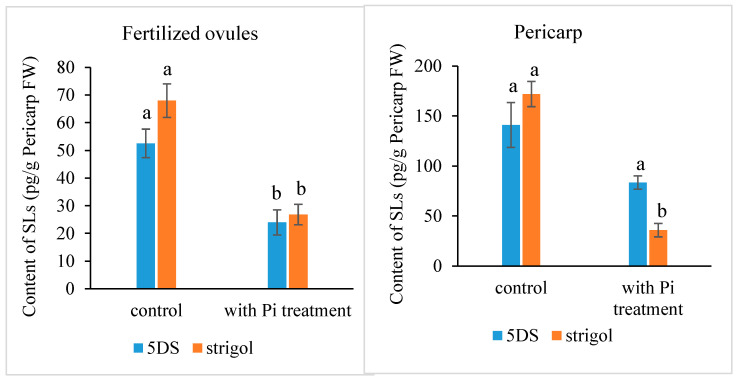
Strigolactone content in the fertilized ovule and the pericarp in the *Xanthoceras* plants under phosphate (Pi)-deficient conditions (control) and with Pi treatment. The values are the means of three biological replicates with standard error bars. Different letters represent significant differences at *p* < 0.05 (ANOVA, Tukey’s HSD test). 5DS: 5-deoxystrigol.

**Figure 3:**
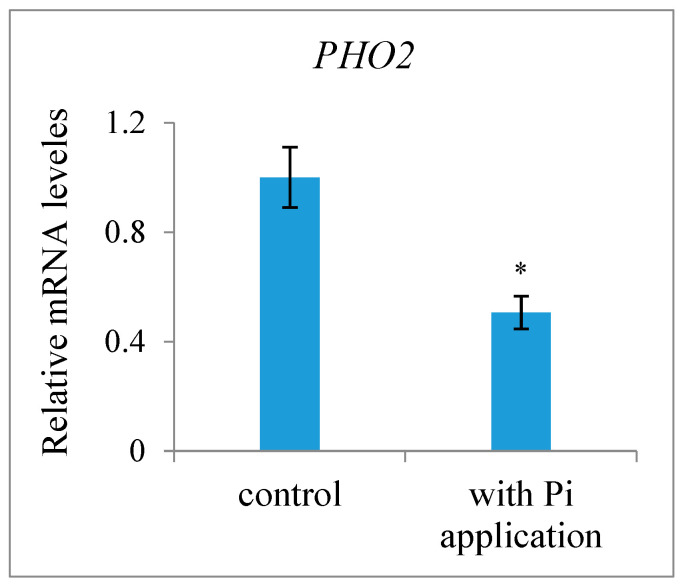
Effect of exogenous phosphate (Pi) application on expression level of the Pi starvation-induced gene *PHO2* in fertilized ovules of the *Xanthoceras* plants without Pi application and with Pi application, which were normalized to the expression level of *actin-2* as a reference. The values are the means of five biological replicates with standard error bars. Asterisks indicate significant difference at *p* < 0.05 (ANOVA, Tukey’s HSD test).

Most SLs have been identified and quantified in root exudates and extracts, whereas studies for SL identification and quantification in other tissues are rare. Here, we determined the presence and level of endogenous SLs in early developing seeds and pericarps using LC-MS/MS and compared the differences between plants with or without an exogenous Pi application treatment. The results indicated that the fertilized ovules and early developing pericarps produce minute amounts of two strigolactones: 5-deoxystrigol and strigol (Figure 1). The contents of 5-deoxystrigol and strigol decreased by 54.39% and 61.03%, respectively, in the fertilized ovules of the plants with an exogenous Pi supply compared with those in untreated Pi-deficient plants (Figure 2). The levels of 5-deoxystrigol and strigol in the young pericarps seemed to change in a similar manner to the fertilized ovules within them (Figure 2). Upon Pi application, the content of strigol was reduced more obviously than that of 5-deoxystrigol in the young pericarps. Pi starvation-induced (PSI) genes are commonly used as markers for the response of plants to low-Pi conditions. *PHOSPHATE2* (*PHO2*), encoding a ubiquitin-conjugating E2 enzyme, is considered a PSI gene in *Xanthoceras* [43]. To confirm that the plants without the Pi application were under low-Pi conditions, we examined the expression of *PHO2* in fertilized ovules. The results showed that transcript levels of *PHO2* were increased in the plants without the Pi application in comparison to the plants with the Pi application (Figure 3). These investigations suggest that Pi availability is associated with the production of SLs in early developing seeds and young fruits.

**Figure 4:**
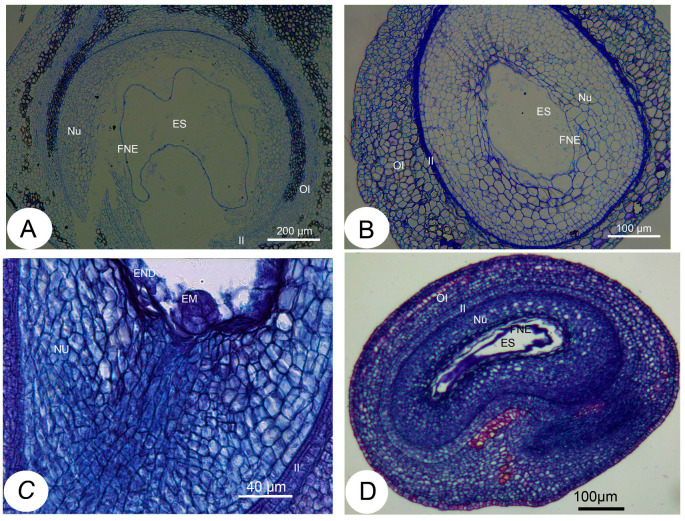
Histological sections of the ovules after fertilization in *Xanthoceras sorbifolium*. (**A**): A portion of a transverse resin section of the normal ovules with low SL levels, showing a rapidly expanding embryo sac and a normally developing syncytial endosperm; (**B**): A portion of a transverse resin section of the abnormal ovules with high SL contents, showing that development of the embryo sac was arrested and syncytial endosperm degenerated. (**C**): A many-celled proembryo was formed at the micropylar end of embryo sac in the normally developing ovule 18 days after pollination (DAP). (**D**). The longitudinal section of the *XsD14*-silenced ovule 7 DAP, showing delayed degeneration. EM: embryo, END: endosperm, ES: embryo sac, FNE: free nuclear endosperm, II: inner integument, NU: nucellus, OI: outer integument.

To elucidate the effect of SL levels on the development of fertilized ovules, we performed comparative cytological studies on the ovules after fertilization. In the fertilized ovules showing a decreased SL content, the embryo sac volume was more quickly enlarged relative to that of the fertilized ovules with high SL levels (Figure 4A,B). The extent of normal nucellar degeneration coincided with the progression of embryo sac growth in fertilized ovules with low SL levels. In contrast, cells in the nucellus tissue were less likely to collapse, embryo sac growth was retarded or completely ceased, and free endosperm nuclei were lower in number and much further apart from each other in the ovules with a high SL content compared with the ovules with reduced SL levels (Figure 4A,B). The resting zygote collapsed in the fertilized ovules with high SL levels, whereas it remained alive in the fertilized ovules with decreased SL contents. These morphological observations show that the abortion process occurs in fertilized ovules with high SL levels, suggesting roles for SLs in the regulation of ovule development after fertilization.

### 2.2. Effect of Exogenous Application of GR24 and TIS108 on Fruit Set and Early Seed Development

**Figure 5:**
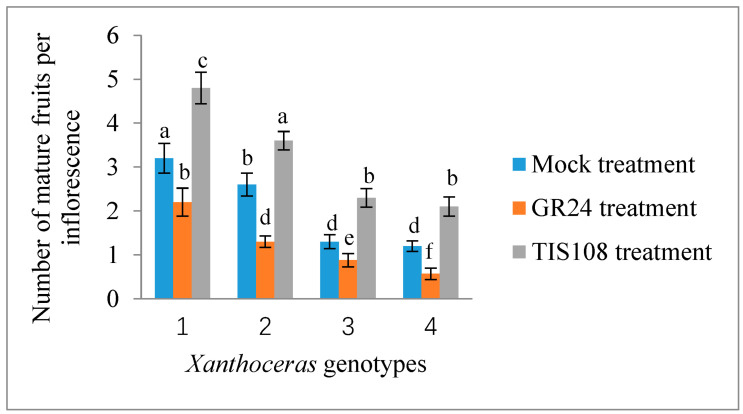
Response of fruit set to treatment with exogenous GR24, a synthetic strigolactone analog, and TIS108, a specific inhibitor of SL biosynthesis, in inflorescences of *Xanthoceras sorbifolium* plants of different genotypes. The same concentration of acetone was used as mock treatment. The values are the means of three biological replicates with standard error bars. Different letters represent significant differences at *p* < 0.05 (ANOVA, Tukey’s HSD test).

**Figure 6:**
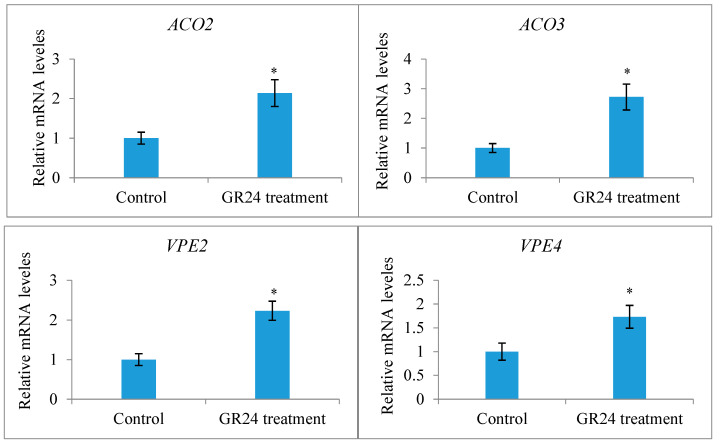
Expression analysis of the genes encoding *Xanthoceras* homologs of ACC oxidase (1-aminocyclopropane-1-carboxylic acid *oxidase*, ACC oxidase, ACO) ACO2 and ACO3 and encoding vacuolar processing enzyme (VPE) VPE2 and VPE4 in the fertilized ovules of the inflorescences with the GR24 or control treatment. The values are the means of three biological replicates with standard error bars. Asterisks indicate significant difference at *p* < 0.05 (ANOVA, Tukey’s HSD test).

Only a terminal racemose inflorescence in a branch can bear functionally female flowers in *X*. *sorbifolium*, an andromonoecious species. Although a single terminal inflorescence may produce as many as 30 functionally female flowers, only a very small percentage of them (usually less than 5%) may set fruits due to various limiting factors, including nutrient deficiency [43]. To determine whether strigolactones affect the fruit set and development of fertilized ovules in *X*. *sorbifolium*, we treated two groups of terminal inflorescences in each genotype with GR24, a synthetic strigolactone analog, and TIS108, a specific inhibitor of SL biosynthesis. The exogenous application of GR24 decreased the number of mature fruits per inflorescence, but there were some differences among the genotypes (Figure 5). Treatment with GR24 accelerated the abortion process of aberrantly developing fruits and the fertilized ovules within them. The inflorescences treated with TIS108 produced more mature fruits than those in the mock treatment. Treatment with TIS108 delayed the abortion process of the fertilized ovules in the aberrantly developing fruits. We examined the expression levels of the genes encoding *Xanthoceras* homologs of ACC oxidase (1-aminocyclopropane-1-carboxylic acid *oxidase*, ACO) ACO2 and ACO3, key enzymes in ethylene biosynthesis [44], in the fertilized ovules of the inflorescences with the GR24 treatment. The results indicated that the GR24 treatment increased the transcript levels of these two *ACO* genes (Figure 6), suggesting the promotion of ethylene biosynthesis. Additionally, an RT-PCR analysis showed that the ovules treated with GR24 had higher expression levels of genes encoding vacuolar processing enzymes (VPEs), which are executors of vacuole-triggered programmed cell death (PCD), including VPE2 and VPE4 [45]. This suggests that ethylene-mediated PCD may be the cause of the cell degeneration in the GR24-treated ovules. All of these findings point to the possibility that SLs influence ovule abortion following fertilization in *Xanthoceras*.

### 2.3. Genome-Wide Identification of SL Biosynthesis Genes and Their Expression in Normally and Abnormally Developing Ovules after Fertilization

To determine whether the morphological changes in the fertilized ovules mentioned above are associated with the extensive reprogramming of gene expression involved in SL biosynthesis and signaling pathways, next-generation RNA sequencing was performed on the mRNAs obtained from normally (with low levels of SLs) and abnormally (with high levels of SLs) developing ovules 10 DAP, which were collected from the plants with the exogenous Pi supply and the ones without the Pi treatment (under Pi deficiency conditions), respectively [43]. Protein sequences of CCD7 (AT2G44990) and CCD8 (AT4G32810) in Arabidopsis were used to search against the *Xanthoceras* genome database (http://gigadb.org/dataset/100606, accessed on 14 May 2019) and the transcriptomic database (this study) using the BLAST tool. Subsequently, each subject sequence was blasted against the NCBI (https://blast.ncbi.nlm.nih.gov/, accessed on 23 January 2021) database to confirm that it contained the conserved domain REP65 or PLN02258 [46]. As a result, we identified a total of 19 candidate CCD family genes, including sixteen members of the CCD subfamily and three members of the 9-cis epoxycarotenoid dioxygenase (NCED) subfamily (Table S1). A phylogenetic tree was constructed using the protein sequences of CCDs and NCEDs from *Xanthoceras*, Arabidopsis, rice, and *Forsythia suspensa*. The results showed that the CCD family proteins were divided into two clades: CCD and NCED (Figure S1).

The transcriptomic analysis indicated that 13 of the 19 CCD genes were expressed (FPKM > 0.1) in the fertilized ovules (Table S1). Of these, nine genes showed differential gene expression between the normal and abnormal ovules (log2 fold change ≥ 1.5 or ≤−1.5; *p* ≤ 0.05), and six genes (including *XsCCD8*) were upregulated in the abnormal ovules. Among the identified CCD family genes, all three *NCED* genes showed significantly increased gene expression in the abnormally developing ovules compared with the normally developing ovules. One of these *NCED* genes, termed *XsNCED3-2* (*EVM0019606*), showed an extremely high abundance of transcripts in both abnormal (FPKM = 645.27) and normal ovules (FPKM = 136.97) (Table S1).

Using the Arabidopsis D27, MAX1, and LBO protein sequences as queries, we identified 4 D27, 19 MAX1, and 16 LBO homologs in the *Xanthoceras* genome, respectively (Table S1). The transcriptomic analysis revealed that 4 *D27*, 16 *MAX1*, and 13 *LBO Xanthoceras* homolog genes were expressed in the fertilized ovules (FPKM > 0.1). Among them, 2 *D27*, 6 *MAX1*, and 12 *LBO* genes were differentially expressed between the normally developing ovules and aborting ovules (Table S1).

**Figure 7:**
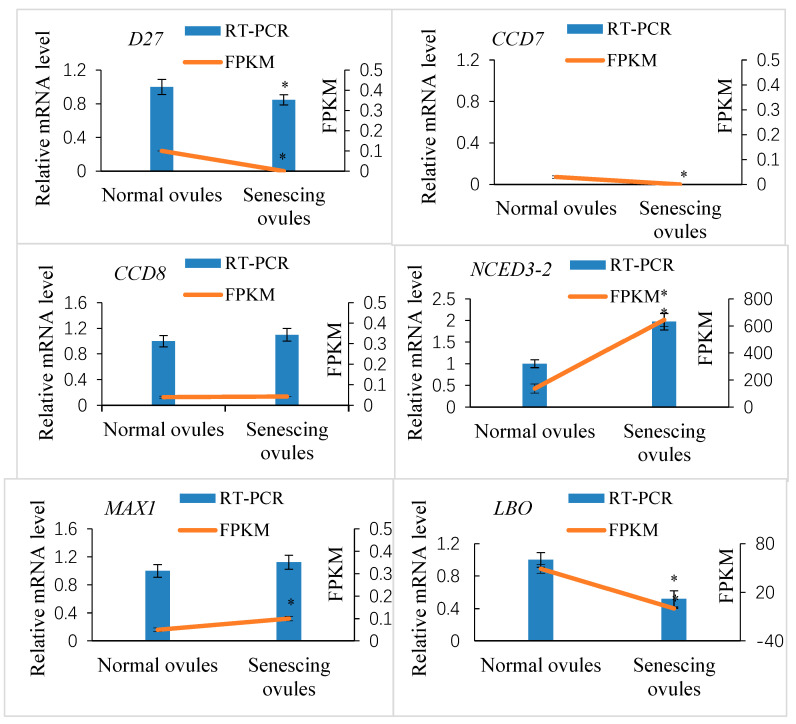
Quantitative real-time PCR (qRT-PCR) verification of expression of the selected SL biosynthesis-related candidate genes in *Xanthoceras* fertilized ovules and comparisons between the qRT-PCR and RNA-Seq data analysis. The *CCD7* gene was not expressed at detectable levels in the qRT-PCR analysis. The values are the means of three biological replicates with standard error bars. Asterisks indicate significant difference at *p* < 0.05 (ANOVA, Tukey’s HSD test).

To validate the quantification of the transcript levels of *Xanthoceras* genes obtained from the RNA-seq approach, the relative gene expression of six selected SL biosynthesis-related candidate genes, including *D27*, *CCD*, *NCED*, *MAX1*, and *LBO*, was measured by quantitative RT-PCR (Figure 7). As shown in the presented data, there was agreement between the results of the qPCR and RNA-seq tests for all six candidate genes examined.

### 2.4. Genome-Wide Identification of Genes Related to SL Perception and Signaling and Their Expression in Fertilized Ovules

**Figure 8:**
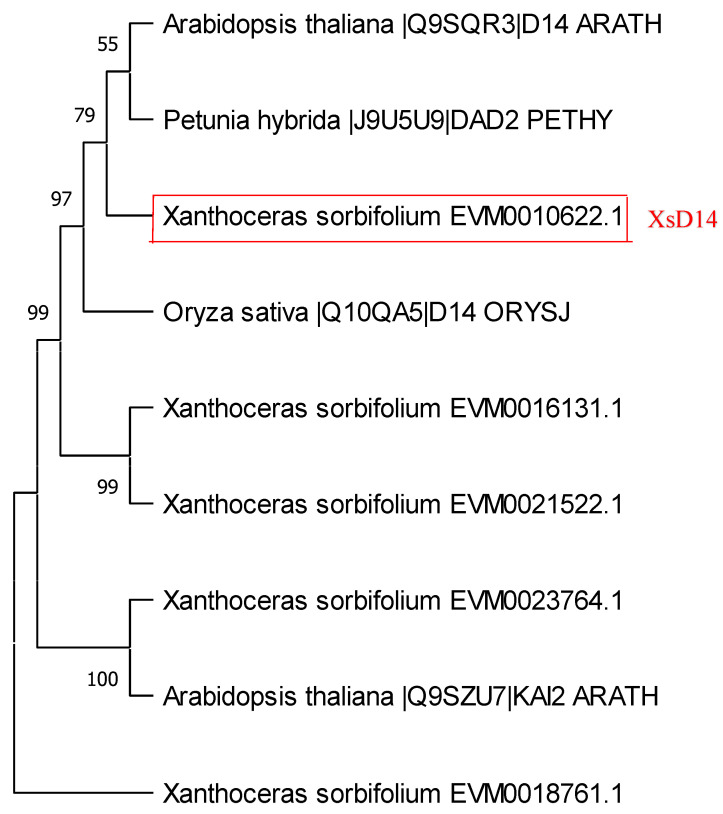
Phylogenetic analysis of DWARF14 (D14) homologs. Maximum likelihood and Bayesian inference phylogenies were produced from an alignment of D14 hydrolases from Arabidopsis, rice, petunia, and *Xanthoceras*. A bootstrap analysis was performed using 1000 replications.

The perception and signaling pathways of SLs in rice and Arabidopsis involve three highly conserved components: D14/AtD14, D3/MAX2, and D53/SMXL6/7/8 [47]. The *D14* gene, first characterized in rice [26], encodes a protein of the α/β hydrolase superfamily with a strictly conserved Ser-His-Asp catalytic triad necessary for hydrolase activity. We used the protein sequence of rice D14 (Os03g0203200) as a query to search for D14 homologs in the *Xanthoceras* genome database. As a result, five *Xanthoceras* D14 homologs (EVM0010622, EVM0016131, EVM0023764, EVM0021522, and EVM0018761) were identified (Table S2). The sequences of these proteins share the putative hydrolase catalytic triad of Ser-Asp-His residues (Figure S2). A phylogenetic analysis of D14 proteins from rice, Arabidopsis, petunia, and *Xanthoceras* indicated that the *Xanthoceras* protein EVM0010622.1 was in the same clade as D14, AtD14, and DAD2 (Figure 8). EVM0010622.1 was the most similar to AtD14, sharing 81.4% of its protein sequence identity; therefore, we designated EVM0010622.1 as XsD14.

**Figure 9:**
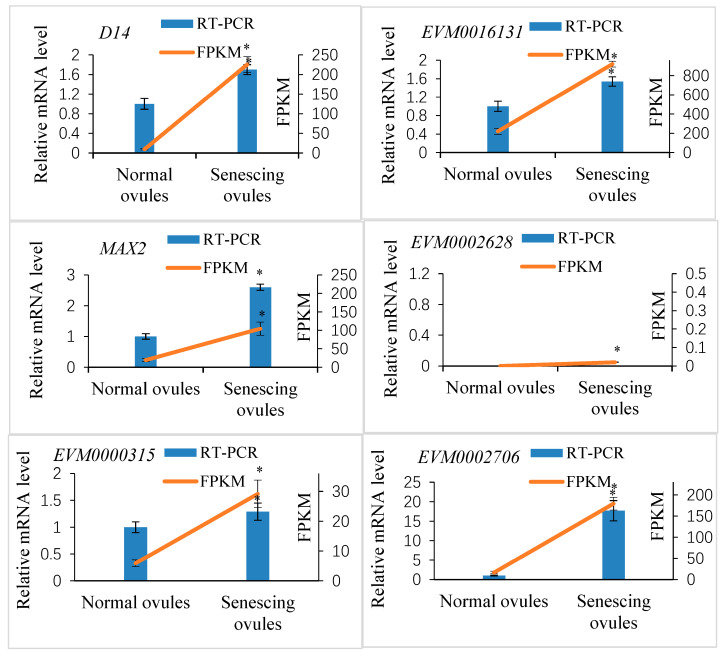
Quantitative real-time PCR (qRT-PCR) verification of expression of the selected SL perception- and signaling-related candidate genes in *Xanthoceras* fertilized ovules and comparisons between the qRT-PCR and RNA-seq data. The values are the means of three biological replicates with standard error bars. Asterisks indicate significant difference at *p* < 0.05 (ANOVA, Tukey’s HSD test).

A transcriptomic analysis indicated that four putative *Xanthoceras D14* homologous genes were expressed in the fertilized ovules, of which *EVM0016131* and *XsD14* showed very high levels of transcription in the aborting ovules (FPKM = 919.2 and FPKM = 225.42, respectively). Among these four genes, three were significantly differentially expressed between the aborting and normally developing ovules (Table S2). The *EVM0016131* and *XsD14* genes were upregulated in the aborting ovules, whereas *EVM0021522* was upregulated in the normally developing ovules. To validate the expression profile of the *Xanthoceras D14* homologous genes, we selected two differentially expressed genes, *XsD14* and *EVM0016131*, for our qRT-PCR analysis. As shown in Figure 9, these two genes showed similar expression patterns to those observed in the RNA-seq data.

Four putative *Xanthoceras* MAX2 homologs (EVM0023678, EVM0002628, EVM0020331, and EVM0007925) were identified with BLASTP searches against the genomic database of *Xanthoceras* using the *Arabidopsis* MAX2 (AT2G42620) protein sequence as a query (Table S2). The EVM0023678 protein was most similar to AtMAX2; therefore, we designated EVM0023678 as XsMAX2, which was 63.7%, 60.7%, and 45.5% identical at the amino acid level to the MAX2 proteins from *P*. *hybrida*, *A*. *thaliana*, and *O*. *sativa* subsp. *japonica*, respectively. The transcriptome analysis showed that the *EVM0002628* and *EVM0020331* genes were not expressed in the fertilized ovules, whereas the *XsMAX2* gene showed very high transcript levels and was significantly upregulated in the aborting ovules compared with the normally developing ovules (Table S2). The results were confirmed by a qRT-PCR analysis of the expression of two selected *MAX2* homologs (Figure 9).

Six putative *Xanthoceras* SMXL homologs were identified by BLASTP against the *Xanthoceras* genome database and the transcriptomic database (this study) using the AtSMXL6 (AT1G07200) protein sequence from Arabidopsis. A phylogenetic analysis of D53 in rice and SMXL proteins in Arabidopsis, pea, and *Xanthoceras* indicated that the *Xanthoceras* protein EVM0012767 was in the same clade as AtSMXL8; therefore, we designated EVM0012767 as XsSMXL8 (Figure S3). This study did not determine the relationship between the other four *Xanthoceras* SMXL homologs and Arabidopsis AtSMXL6 and AtSMXL7. The protein sequence analysis revealed that the XsSMXL8 protein contains an RGKT motif, which is highly conserved in rice D53, Arabidopsis AtSMXL6/7/8, and pea PsSMXL8 and considered to be essential for SL-mediated D53/SMXL protein degradation [48]. The transcriptomic analysis indicated that all six *Xanthoceras SMXL* homolog genes were expressed in the early developing seeds. Among them, four showed significantly differential expression between the degenerating and normally developing ovules after fertilization, and all were upregulated in the aborting ovules compared with the normal ovules. The qRT-PCR analysis of two selected DEGs validated the differences in transcript abundance from the RNA-seq analysis (Figure 9).

### 2.5. Genome-Wide Identification of Xanthoceras Invertase Genes and Crosstalk between Sugar and Strigolactone in the Development of Fertilized Ovules

Based on their pH optima, invertases are classified into acid invertase (A-Inv) and alkaline/neutral invertase (A/N-Inv) [49]. According to their subcellular localization, acid invertases are further subdivided into cell-wall-bound invertase (CWIN) and vacuolar invertase (VIN). Alkaline/neutral invertases have different subcellular localizations, including cytosol, mitochondria, chloroplast, and nuclei. Based on the sequences of six cell wall invertase (AtcwInv1–6), two vacuolar invertase (AtvaInv1–2), and eleven neutral/alkaline invertase (At-A/N-InvA-K) proteins from Arabidopsis and a BLASTP search, we identified six cell wall, two vacuolar, and eight alkaline/neutral invertase homologs in the *X*. *sorbifolium* genome (Table S3). The alignment analysis of the deduced protein sequences of the *Xanthoceras* CWINs and VINs indicated that they contain a β-fructofuranosidase motif (NDPNG/A), an RDP motif, and a cysteine catalytic domain (WECP/VD), as well as four putative enzyme active site residues [49] (Figure S4). A valine residue in the ‘WEC-P/V-D’ box was found for two vacuolar invertases, whereas six cell wall invertases were characterized by the presence of a proline residue (Figure S4). Twelve conserved domains, which have been identified in rice A/N-Invs [49], could be detected in all eight *Xanthoceras* alkaline/neutral invertases (Figure S5). Consistently, a phylogenetic analysis showed that the invertase proteins from *Xanthoceras*, *Arabidopsis*, *Litchi* (Sapindaceae), and *Dimocarpus* (Sapindaceae) were clustered into two subgroups: acid invertases and alkaline/neutral invertases (Figure S6). The acid invertase clade contained cell-wall- and vacuole-targeted subgroups.

**Figure 10:**
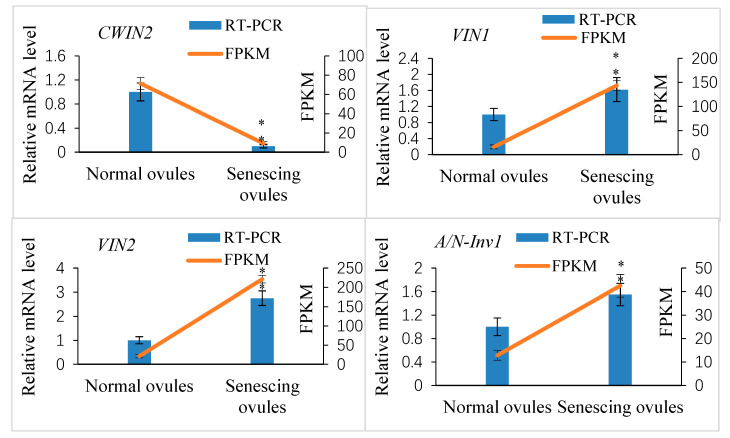
Quantitative real-time PCR (qRT-PCR) verification of expression of the selected invertase candidate genes in *Xanthoceras* fertilized ovules and comparisons between the qRT-PCR and RNA-seq data. The values are the means of three biological replicates with standard error bars. Asterisks indicate significant difference at *p* < 0.05 (ANOVA, Tukey’s HSD test).

Our transcriptome data analysis revealed that 12 putative invertase genes were actively expressed in normally developing ovules at 10 DAP (FPKM ≥ 10; Table S3). Of these, four genes showed significantly differential expression between the abnormally and normally developing ovules (log2-fold change ≥1.5 or ≤−1.5; *p* ≤ 0.05). The cell wall invertase gene *XsCWIN2* (*EVM0004408*) was upregulated in the normally developing ovules, whereas two vacuolar invertase genes, *XsVIN1* (*EVM0014291*) and *XsVIN2* (*EVM0018616*), and an alkaline/neutral invertase gene, *XsA/N-Inv1* (*EVM0006730*), were downregulated in the normally developing ovules. The transcript abundance of these four DEGs was validated by a qRT-PCR analysis (Figure 10).

Based on the data above, we hypothesized that strigolactone may regulate early seed development by interacting with sugars in *Xanthoceras* and that the crosstalk between sugar and strigolactone may occur via sucrose metabolic processes. To preliminarily test this idea, we measured the endogenous content of soluble sugars in various developmental stages of the ovules after pollination using LC-MS/MS. The results indicated that the sucrose levels rose steadily in the normally developing ovules before 20 DAP and then increased dramatically during the formation and early development of cotyledons between 20 and 28 DAP (Table 1). The embryo at the middle stage of development between 40 and 48 DAP contained high levels of sucrose. The fructose and glucose contents also increased progressively before 13 DAP, while the free nuclei of the developing endosperm divided rapidly and accumulated on the embryo sac periphery. The ratio of hexose to sucrose reached the highest value at 13 DAP and then decreased following the formation of many-celled proembryos at the micropylar end of the embryo sac (Figure 4C). Oligosaccharide (raffinose) was not detected in the fertilized ovules until 28 DAP, and its content was high in the embryo at 48 DAP. Very high levels of sucrose and relatively low levels of hexose were found in the aborting ovules with a high strigolactone content at 12 DAP, whereas only traces of sucrose could be detected in the aborting ovules at 20 DAP (Table 1).

**Figure 11:**
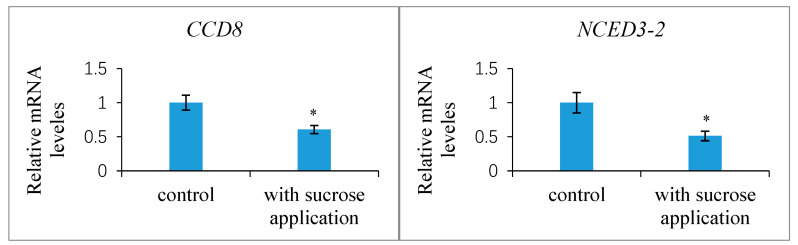
Expression levels of *CCD8* and *NCED3-2* genes in the *Xanthoceras* fertilized ovules with sucrose application and control treatment, respectively, relative to the housekeeping gene actin-2. The values are the means of three biological replicates with standard error bars. Asterisks indicate significant difference at *p* < 0.05 (ANOVA, Tukey’s HSD test).

**Figure 12:**
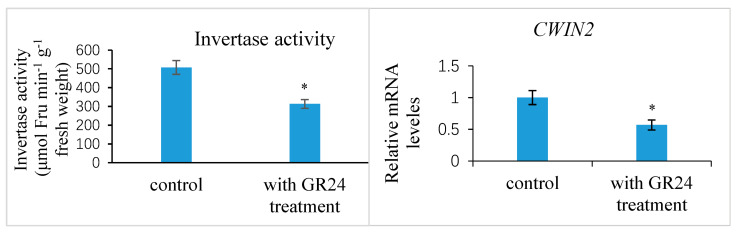
Invertase activity and relative expression levels of *CWIN2* gene in the *Xanthoceras* fertilized ovules with a synthetic strigolactone analog GR24 treatment and control treatment, respectively. The values are the means of three biological replicates with standard error bars. Asterisks indicate significant difference at *p* < 0.05 (ANOVA, Tukey’s HSD test).

Further experimental studies revealed that the exogenous treatment of the ovaries at 1 DAP with 100 mM sucrose reduced the transcript levels of the putative strigolactone biosynthesis genes *CCD8* and *NCED3-2* in the ovules at 7 DAP compared to the control, indicating the likelihood that the strigolactone decreased (Figure 11). This exogenous sucrose application either delayed the abortion process of early aberrantly developing fruits and the fertilized ovules within them or partially restored the development of these fruits and ovules. Additionally, we assessed the expression of the *CWIN2* gene and the invertase enzyme activity in ovules 9 DAP treated with exogenous strigolactone (GR24) 1 DAP. The results showed that both the invertase activity and *CWIN2* transcript amounts in the fertilized ovules with the GR24 treatment were lower than those in the control ovules (Figure 12), in parallel with the low accumulation of glucose and fructose in the aborting ovules, suggesting a potential inhibitory effect of strigolactone on invertase activity.

### 2.6. Silencing of the XsD14 Gene Promoted Early Seed Development

**Figure 13:**
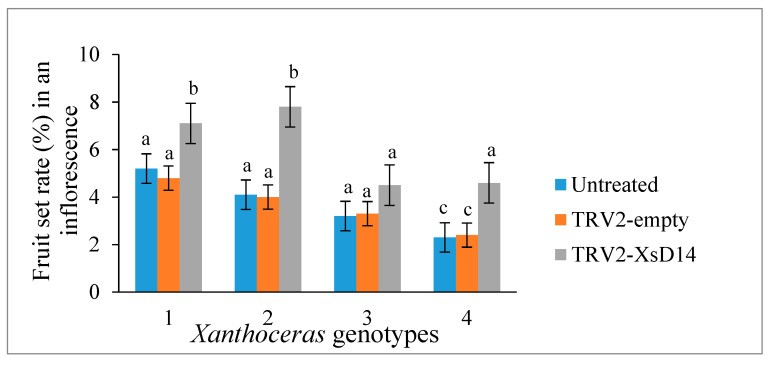
Effect of VIGS-mediated *XsD14* gene silencing on fruit set in terminal inflorescences of 4 *Xanthoceras sorbifolium* genotypes. The values are the means of five biological replicates with standard error bars. Different letters represent significant differences at *p* < 0.05 (ANOVA, Tukey’s HSD test).

**Figure 14:**
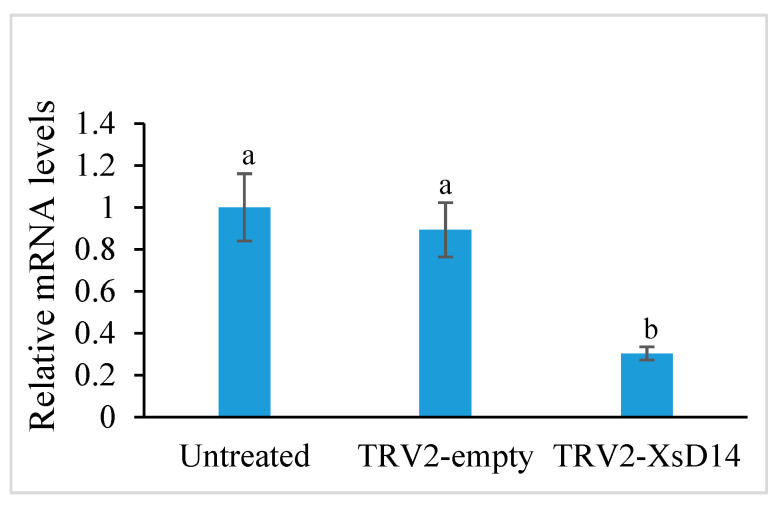
Expression levels of *XsD14* gene in the fertilized ovules with VIGS-mediated *XsD14* gene silencing treatment and control treatment in relation to the expression levels of actin-2. The values are the means of three biological replicates with standard error bars. Different letters represent significant differences at *p* < 0.05 (ANOVA, Tukey’s HSD test).

**Figure 15:**
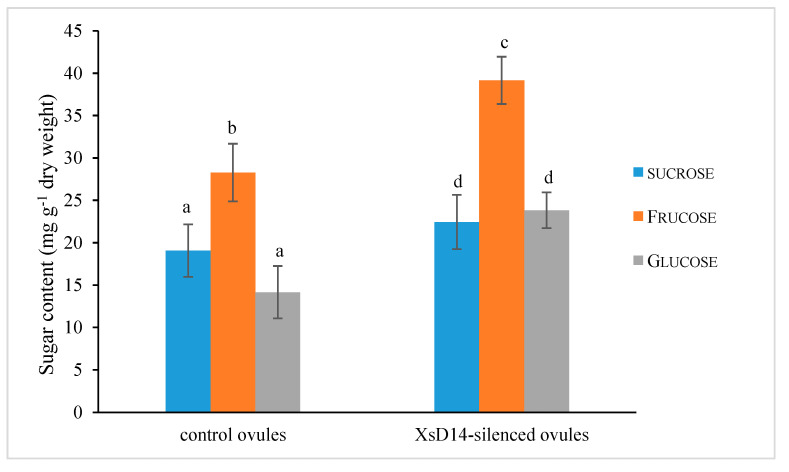
Soluble sugar content in the fertilized ovules with VIGS-mediated *XsD14* gene silencing treatment and control treatment. The values are the means of three biological replicates with standard error bars. Different letters represent significant differences at *p* < 0.05 (ANOVA, Tukey’s HSD test).

As mentioned previously, the *XsD14* gene showed very high transcript abundance in the fertilized ovules and was upregulated in the aborting ovules, so we further characterized the role of this gene during early seed development. TRV-based VIGS has been successfully applied to induce the silencing of endogenous genes in young fruits and fertilized ovules in *X*. *sorbifolium* [43]. The present study also used this approach to silence the expression of the *XsD14* gene in young fruits and fertilized ovules by injecting an *Agrobacterium tumefaciens* suspension containing the pTRV2-*XsD14* vector into terminal inflorescences 3 d before anthesis. Our phenotypic observations showed that pTRV2-*XsD14* infiltration led to higher fruit retention in the inflorescences than the pTRV2-empty vector control treatment, but there were differences between genotypes in terms of the silencing effects (Figure 13). The qRT-PCR analyses indicated that *XsD14* mRNA levels were significantly reduced in the fertilized ovules within the pTRV2-*XsD14*-infiltrated fruits, compared with those in the control (Figure 14). The development of the fertilized ovules was arrested 7 DAP in untreated aberrantly developing fruits, whereas the silencing of the *XsD14* gene resulted in a delay in the arrest and abortion process of the fertilized ovules. The results of a cytological investigation and sugar quantification showed that the *XsD14*-silenced ovules had a larger hexose content (Figure 15) and more durable proliferation of endosperm free nuclei (Figure 4D) than the control ovules.

## 3. Discussion

Most studies on SL biosynthesis and signaling have focused on roots and shoots, but little is known about whether SLs are produced in early developing seeds and about their roles in ovule development after fertilization [1,47]. This study revealed that SLs were also generated in early developing seeds and that the aborting ovules after fertilization contained a high accumulation of SLs during the early stages of endosperm development in *X*. *sorbifolium* plants under low-Pi conditions. Enhanced SL biosynthesis was associated with higher expression levels of *PHO2*, a PSI gene, in the plants exposed to Pi starvation than in the plants with the Pi application. The inorganic form of phosphorus (phosphate, Pi) that is available to plants is a severely limiting factor for the fruit and seed production of *Xanthoceras* due to its low availability in most soils [43]. The requirement of *Xanthoceras* plants for phosphorus nutrients may be quite high during early seed development because of the active proliferation and growth of the syncytial endosperm and maternal tissues, including the nucellus and integuments, followed by the rapid expansion of the large embryo sac after fertilization [43,50]. We assume that the fertilized ovules in *X*. *sorbifolium* are the most sensitive to Pi deficiency. Plants have evolved elaborate mechanisms for adapting to low-Pi conditions and for maintaining cellular Pi homeostasis [5,12,51]. Many lines of evidence support the involvement of hormone-dependent signaling pathways, including SL pathways, in Pi starvation responses that lead to increased Pi uptake and reallocation within the plant [12,52]. Based on the present and previous studies, we propose that the development of fertilized ovules during Pi deficiency might be influenced by SL signaling and could be utilized during the process of Pi starvation responses as a way to fine-tune the adaptations of *Xanthoceras* plants to environmental stress conditions.

Here, we showed that there was an obvious difference in the progression of abortion between ovules treated with GR24 and those with the control treatment. The abortion process of the fertilized ovules in the aberrantly developing fruits was accelerated by the treatment with GR24; in contrast, the ovule abortion process was delayed by the treatment with TIS108, a triazole derivative that inhibits SL biosynthesis. The accelerated abortion process of the ovules with the GR24 treatment was associated with the degeneration of free endosperm nuclei and rapid reduction of the embryo sac. Our previous study identified a number of ethylene biosynthesis and signaling genes whose transcript abundances were significantly elevated in aborting ovules after fertilization [43]. The present study indicated that the GR24 treatment significantly induced the expression of *Xanthoceras* homologs of *ACO* genes, key components involved in ethylene biosynthesis, in fertilized ovules. This was consistent with findings of Lee and Yoon (2020) [53], whose treatment of etiolated Arabidopsis seedlings with GR24 increased the transcript levels of *ACO* genes, thereby enhancing ethylene biosynthesis. Our studies imply that SLs possibly act as abortion signals of fertilized ovules in *X*. *sorbifolium* and that potential crosstalk between SLs and ethylene may be involved in the control of ovule abortion after fertilization.

Our transcriptome analysis identified a total of 62 putative *Xanthoceras* homologs of the proposed SL biosynthesis genes in the *Xanthoceras* genome, including the *D27*, *CCD*, *MAX1*, and *LBO* genes. *CCD7*/*MAX3* and *CCD8*/*MAX4* have been shown to be expressed mainly in roots and lower stems in various examined species [15,46,54,55]. We found that several *CCD* family genes, including *CCD8* and three *NCED* subfamily genes, were also expressed in the early developing seeds, of which some genes showed increased transcript levels in the aborting ovules after fertilization. The most similar *Xanthoceras* homolog to *AtMAX1* was only weakly expressed in the fertilized ovules, but several other *MAX1* homologues showed abundant transcripts and differential expression between the normal and aborting ovules. The expression patterns observed for multiple SL biosynthesis genes seem to support their activities in determining the amount of SLs (including 5-deoxystrigol and strigol) accumulated in early developing seeds, in which their upregulated expression might increase the SL levels in the aborting ovules after fertilization. These transcripts likely contribute to the key traits required to enhance the abortion process of fertilized ovules in the plant.

Substantial evidence has established that the D14 α/β-fold hydrolase functions as an SL receptor and is required for the perception of the SL signal in petunia, rice, Arabidopsis, pea, and tomato [47]. The *D14* gene was transcribed at high levels in rosette and cauline leaves and at lower levels in axillary buds, inflorescences, stems, and roots in Arabidopsis plants [56]. In *Brassica napus*, CRISPR/Cas9-mediated knockout lines of the genes encoding BnD14 showed the characteristic feedback upregulation of *CCD7* and *CCD8* transcripts [57]. The lack of BnD14 function resulted in a prolific branching phenotype and an increase in total flower and total pod weight per plant. Our study found that the *D14* gene was also expressed in fertilized ovules in which *Xanthoceras* homologs of *MAX2* and SLs coincide. This is consistent with the proposed interaction between D14 and MAX2 in the presence of SLs [29], suggesting that the SL response takes place in the fertilized ovules where these factors coincide. We used a VIGS approach to characterize *XsD14* functions during reproductive processes in *X*. *sorbifolium*. The results demonstrated that *XsD14* gene silencing could alleviate fruit abortion and delay the abortion process of fertilized ovules, suggesting an important role of this gene in regulating the early development of fruits and seeds.

*Xanthoceras sorbifolium* follows a nuclear-type endosperm development, in which the endosperm initially develops as a syncytium, and cellularization is triggered after the formation of globular embryos [41]. The fertilized egg cell does not start to divide until 11 d after pollination, unlike the central cell, which quickly undergoes many mitotic cycles after fertilization to form the syncytial endosperm. Zygote division is a crucial developmental transition, which, in the case of failure, leads to ovule abortion [58]. It is likely that the syncytial endosperm produces structural and signaling substances such as sugars, which could activate the resting zygote and act as a nutritive component supporting early embryo growth. Fruit growth and ovule development after fertilization show great plasticity in *X*. *sorbifolium*, as they are regulated by pollen sources and nutrient availability [50]. Under low-nutrient conditions, up to 70% of the young fruits are aborted within 4 to 11 d following pollination. On average, the endosperm develops for 3 to 8 days before ovule abortion and the zygote degenerates during this period. The early collapse of the zygote can be used for identifying abortive ovules. By applying a sucrose treatment, the growth of young fruits and fertilized ovules within them is partially restored from an early abnormality. When nutrients, such as sugar and Pi, are limiting, increased SLs possibly inhibit fruit development to avoid the nutrient-demanding growth of the aborting fruits and fertilized ovules within them, thereby reducing sink tissue. We assume that the SL-mediated promotion of fruit and ovule abortion in *X. sorbifolium* might allow for a reallocation of resources to healthy fruits or other parts of the plant under nutrient-deficient conditions.

We found that hexoses (glucose and fructose) accumulated at high concentrations in fertilized ovules during the syncytial endosperm development, whereas sucrose increased during the mid- and late-development stages. High ratios of hexose and sucrose during early seed development might promote the proliferation of endosperm free nuclei and syncytial endosperm development in *X*. *sorbifolium*. When the ratio of hexose to sucrose levels decreased significantly, the growth of the fertilized ovules might be arrested and their abortion process would be initiated. The development of the fertilized ovules might be positively correlated with the hexose content but negatively correlated with SL levels. SL accumulation caused changes in the ratio of hexose to sucrose during ovule degeneration, suggesting the possibility that SLs may control the development of ovules after fertilization through their effects on the activity of invertases. The invertase-mediated release of hexoses is likely critical for appropriate carbon partitioning and normal seed development in *X*. *sorbifolium*.

## 4. Materials and Methods

### 4.1. Plant Materials

Seven-year-old *Xanthoceras sorbifolium* trees that were used in this study were grown at the experimental farm of the Institute of Botany, Chinese Academy of Sciences (Xiangshan, Beijing, China). Functional female flowers were pollinated on the first day of anthesis. Ovules were isolated from the ovaries at various developmental stages after pollination. Isolated ovules were flash frozen in liquid nitrogen and stored at −80 °C until further use. For cytological studies, some collected ovules were immediately fixed with FAA (formaldehyde acetic acid) solution containing 50% (*v*/*v*) ethanol, 5% (*v*/*v*) acetic acid, and 3.7% (*w*/*v*) formaldehyde for at least 24 h and then stored at 4 °C.

### 4.2. Histological Analysis

Fixed ovules were dehydrated, infiltrated, and embedded in either Paraplast Plus or Spurr resin. Paraplast Plus sections were cut to 6–10 μm using a steel knife and stained with safranin and fast green. Resin sections were cut to be 1–1.5 μm thick with a diamond knife and stained with 0.5% toluidine blue O in 0.1% sodium carbonate (pH 11.1) for general observations. Stained sections were observed with a Carl Zeiss microscope (Carl Zeiss Microscopy GmbH, Jena, Germany).

### 4.3. Strigolactone Analysis

Frozen ovules were homogenized to a fine powder under liquid nitrogen, and SL extraction was performed using 1 mL acetone containing d1-*epi*-5DS (100 pg) at −20 °C overnight. After the samples were filtered, and the filtrates were dried under nitrogen gas and then dissolved in 10% acetone. The extracts were loaded onto Oasis HLB (Waters Corporation, Micromass, TA, USA) and Sep-Pak Silica (Waters, Micromass, TA, USA) cartridges for purification and then washed with water, eluted with acetone, and dried under nitrogen gas. The SL-containing fraction was eluted with acetone and then evaporated until dry. The sample was re-dissolved in 50% acetonitrile (acetonitrile:water = 50:50, *v*:*v*) and filtered through a filter for liquid chromatography–tandem mass spectrometry (LC-MS/MS) analysis. SLs were analyzed using a high-pressure liquid chromatography (HPLC) Q-Trap-MS/MS in MRM (multiple reaction monitoring) mode.

### 4.4. Soluble Sugar Analysis

Ovules at various developmental stages after pollination were sampled for soluble sugar analysis. The samples were lyophilized with a freeze drier (SYHX, Beijing, China) and subsequently ground to a powder in liquid nitrogen. Sugars were extracted with an 80% aqueous methanol (*v*/*v*) solution and centrifuged for 15 min at 20 °C. The supernatant was collected and evaporated in a centrifugal vacuum evaporator (CV100-DNA, Aijimu, Beijing, China). Chloroform and distilled water were added to the samples and then centrifuged. The supernatant was dried by a vacuum concentrator at room temperature. The dried samples were oximated with methoxyamination reagent at 37 °C for 2 h. Then, the silylation reagent, MSTFA, was added to each oximated sample. The mixture was incubated at 60 °C for 30 min. Finally, the derivatized samples were transferred to glass vials for GC-MS analyses using an Agilent 7890A GC system equipped with an Agilent 7693 autosampler and Agilent 5975C-inert MSD with Triple-Axis Detector (Agilent, Atlanta, GA, USA). Three replicates were used for each sample at the same stages.

### 4.5. Invertase Enzyme Assay

Ovules were ground to powder in liquid nitrogen, followed by the addition of 150 mM Tris-HCl (pH 8.0) extraction buffer containing 2 mM EDTA, 10 mM MgCl_2_, 0.2% (*v*/*v*) 2-mercaptoethanol, 0.1 mM phenylmethyl sulfonyl fluoride, 1 mM benzamidine, and 10 mM ascorbic acid. Homogenates were centrifuged at 15,000× *g* rpm for 20 min at 4 °C. The sediments were homogenized with 150 mM Tris-HCl (at pH of 8.0) extraction buffer (the same as above) and subsequently centrifuged at 15,000× *g* rpm for 20 min at 4 °C. The supernatants were desalted twice using Sephadex G-25 (Pharmacia PD-10) and kept on ice until use. The mixture was incubated at 37 °C for 120 min. The desalted extracts and glucose were used as background control and standard, respectively. The reaction was stopped by adding the stop solution containing 1 M sodium potassium tartrate, 1% 3,5-dinitrosalicylic acid, and 0.5 M KOH. The liberated reducing sugars were quantified by measuring the absorbance at 540 nm.

### 4.6. Assays of GR24 and TIS108 Treatment

Concentrations for GR24 at 2.5 μM, an SL analog, and TIS108 at 1.5 μM, an SL biosynthetic inhibitor, were empirically determined. Both compounds were initially dissolved in acetone and volume was then adjusted with double-distilled sterile water. Young fruits at 1 DAP were dipped in the GR24 or TIS108 solution every other day for 6 days of the induction process. Treatments with the same concentration of acetone (diluted with water) were used as the experimental control. Each experiment was performed using three biological replicates.

### 4.7. Assays of Sucrose Treatment

Concentration for sucrose at 100 mM was empirically determined. Ovaries at 1 DAP were dipped in the sucrose solutions every other day over 6 days of the induction process. Effects of sucrose supply on fruit growth and ovule development were compared with mannitol, an osmotic control. Each experiment was performed using three biological replicates.

### 4.8. Phosphorus (P) Applications

Phosphorus application experiments were conducted with seven-year-old trees of 4 *Xanthoceras* genotypes (5 trees per genotype). Either phosphorus was not applied (control) or phosphorus fertilization dose of 150 g P_2_O_5_ per tree was used. Triple superphosphate (46% P_2_O_5_) was used as the source of phosphorus. Applications were realized in the form of a circle (40 cm radius) in front of roots. Plants received P applications 10 days before anthesis.

### 4.9. RNA Extraction, Library Construction, and Illumina Sequencing

Based on histological observations of ovules after fertilization, normal and abnormal ovules were determined and each was sampled for RNA-seq analysis. The total RNA from the samples was extracted using a Plant Total RNA Isolation Kit (Huayueyang, Beijing, China). A total of 6 RNA preparations (3 biological replicates for each sample) were used to increase sequencing coverage. The quantity and quality of extracted RNA were evaluated using a Qubit fluorometer (Invitrogen Inc., Carlsbad, CA, USA) and bioanalyzer 2100 (Agilent Technologies, Santa Clara, CA, USA), respectively. Oligo-dT (Qiagen, Valencia, CA, USA) was used to to isolate mRNA. The mRNA was fragmented using fragmentation buffer. cDNA was then synthesized using the mRNA fragments as templates. The cDNA fragments were purified using Qiaquick PCR Purification Kits (Qiagen, Valencia, CA, USA). The purified cDNA fragments were end repaired, added to poly (A), and ligated to Illumina sequencing adapters. Suitable fragments were selected for the PCR amplification and sequenced using BGISEQ-500 at BGI (Shenzhen, China).

### 4.10. RNA-Seq Data Analysis

Raw RNA-seq reads were filtered using a Perl program to obtain high-quality clean reads by removing low-quality sequences (having more than 20% bases with quality lower than 15 in one sequence), reads with more than 5% N bases (bases unknown), and reads containing Illumina sequencing adapters. The raw RNA-seq data have been deposited in the publicly accessible NCBI Sequence Read Archive (SRA) database under Bioproject PRJNA966773 (https://www.ncbi.nlm.nih.gov/sra/PRJNA966773, accessed on 3 May 2023). The filtered sequence pairs were aligned to the *Xanthoceras sorbifolium* reference genome sequences, using the alignment tool HISAT2 version 2.1.0. The transcript levels of individual transcripts in each sample were normalized as fragments per kilobase of transcript per million mapped reads (FPKM). Differentially expressed genes (DEGs) were identified using DEseq2.

### 4.11. Real-Time qRT-PCR Analysis

Real-time qRT-PCR was used to estimate the accuracy of RNA-seq data. The same RNA samples used for RNA-seq were used for qRT-PCR. RNA was used for synthesis of cDNA with the PrimeScript RT Master Mix Perfect Real Time Kit (Takara, San Jose, CA, USA). Real-time qRT-PCR was performed on the StepOnePLUS^TM^ real-time PCR System (Applied Biosystems, Waltham, MA, USA). The actin gene (*EVM0010329*) was used as an internal reference, and relative expression was calculated using the 2^−△△Ct^ method. Each sample was analyzed using 3 independent biological replicates. All of the primers used in this study are listed in Table S4.

### 4.12. Virus-Induced Gene Silence (VIGS) Assays

A 370 bp cDNA fragment of *XsD14 gene* was amplified using the primers presented in Table S4. The purified PCR products were digested with *EcoRI* and *KpnI* and ligated to pTRV2, resulting in the plasmid TRV2-*XsD14*. The plasmids were sequenced to verify correct insertion of the fragment and were then transformed into *Agrobacterium tumefaciens* strain GV3101. The cultures containing pTRV1 and pTRV2/pTRV2-*XsD14* vector were mixed in a 1:1 ratio, and then 1 mL of culture was injected into the base of an inflorescence axial. Five independent biological replicates were performed for each treatment.

### 4.13. Statistical Analysis

All the data were analyzed statistically using SPSS 25.0 (SPSS Inc., Chicago, IL, USA) software. One-way analysis of variance (ANOVA) test was performed to assess the statistical differences. Data are shown as the means ± standard error of at least three biological replicates, and the mean differences were evaluated by Tukey’s honestly significant difference test at *p* < 0.05.

## 5. Conclusions

After its ovules are fertilized, the *Xanthoceras* plant decides whether or not to proceed with seed development. Signals from the environment, including the Pi nutritional state, and endogenous production, such as metabolite and hormone levels, influence these decisions. The genes and chemicals that participate in this finely tuned network of communication must be identified and characterized. This study identified a total of 69 putative *Xanthoceras* homologs of genes related to SL biosynthesis and signaling in the *Xanthoceras* genome; among them, 37 genes were found to be differently expressed in the fertilized ovules that were aborting compared to the normally developing ovules, suggesting their important roles in seed development and environmental responses. These genes could be ideal candidates for further studies and *Xanthoceras* germplasm genetic engineering in the future. The present study indicated that the production and signaling pathways of SLs are likely correlated with Pi deficiency, invertase activity, and sugar availability in ovules after fertilization. The crosstalk between sugar and strigolactone signals may be an important part of a system that accurately regulates ovule development after fertilization in *X*. *sorbifolium*.

## Figures and Tables

**Table 1:** Soluble sugar content in fertilized ovules and early embryos in *Xanthoceras sorbifolium*. Values (mg g^−1^ dry weight) are the means ± SD of four biological replicates.

Samples	Xylose	Fructose	Glucose	Arabinose	Inositol	Sucrose	Glucose 6-Phosphate	Raffinose
A	0.005 ± 0.001 a	32.741 ± 4.674 a	16.482 ± 4.321 a	0.010 ± 0.003 a	6.838 ± 1.351 a	23.947 ± 4.761 a	0.369 ± 0.071 a	0.000 a
B	0.009 ± 0.002 b	45.481 ± 5.341 a	20.209 ± 3.312 a	0.025 ± 0.006 b	14.806 ± 2.147 b	162.724 ± 14.751 b	0.422 ± 0.123 a	0.000 a
C	0.049± 0.016 a	63.731 ± 6.454 b	22.423 ± 2.261 a	0.061 ± 0.017 c	4.726 ± 1.314 a	4.166 ± 1.531 c	0.798 ± 0.241 b	0.000 a
D	0.020 ± 0.045 c	137.265 ± 8.671 c	59.152 ± 7.251 b	0.022 ± 0.006 b	16.197 ± 2.765 b	27.066 ± 5.141 a	0.569 ± 0.156 a	0.000 a
E	0.023 ± 0.052 c	94.150 ± 9.543 d	34.826 ± 5.432 c	0.045 ± 0.017 d	8.300 ± 1.475 a	41.448 ± 4.751 d	0.121 ± 0.047 c	0.000 a
F	0.042 ± 0.047 a	78.163 ± 7.241 d	59.411 ± 6.423 b	0.019 ± 0.008 b	6.825 ± 1.417 a	204.921 ± 23.241 e	1.685 ± 0.491 d	0.937 ± 0.251 a
G	0.014 ± 0.041 d	69.939 ± 6.653 b	66.181 ± 5.174 b	0.004 ± 0.001 a	13.357 ± 2.433 b	185.235 ± 22.741 b	0.566 ± 0.156 a	3.258 ± 0.753 b

A: normal ovules at 10 days after pollination (DAP); B: normal ovules 13 DAP; C: normal ovules 20 DAP; D: normal ovules 28 DAP; E: normal embryo 48 DAP; F: degenerating ovules 12 DAP; G: degenerating ovules 20 DAP. Values accompanied by different lower-case letters differ significantly (*p* < 0.05) between samples.

## Data Availability

All data generated and used in this study are included in this article and its additional files. The raw RNA-seq data have been deposited in the publicly accessible NCBI Sequence Read Archive (SRA) database under Bioproject PRJNA966773 (https://www.ncbi.nlm.nih.gov/sra/PRJNA966773, accessed on 3 May 2023).

## Supplementary Materials

The following supporting information can be downloaded at: https://www.mdpi.com/article/10.3390/ijms25063276/s1.
