# Supplementary material for: Strigolactones Might Regulate Ovule Development after Fertilization in Xanthoceras sorbifolium
Source: Int J Mol Sci. 2024 Mar 14;25(6):3276. doi: 10.3390/ijms25063276 (PMC10969979; doi:10.3390/ijms25063276)
Supplement: Supplementary file 1 [file ijms-25-03276-s001.zip › Figure S1.pdf]

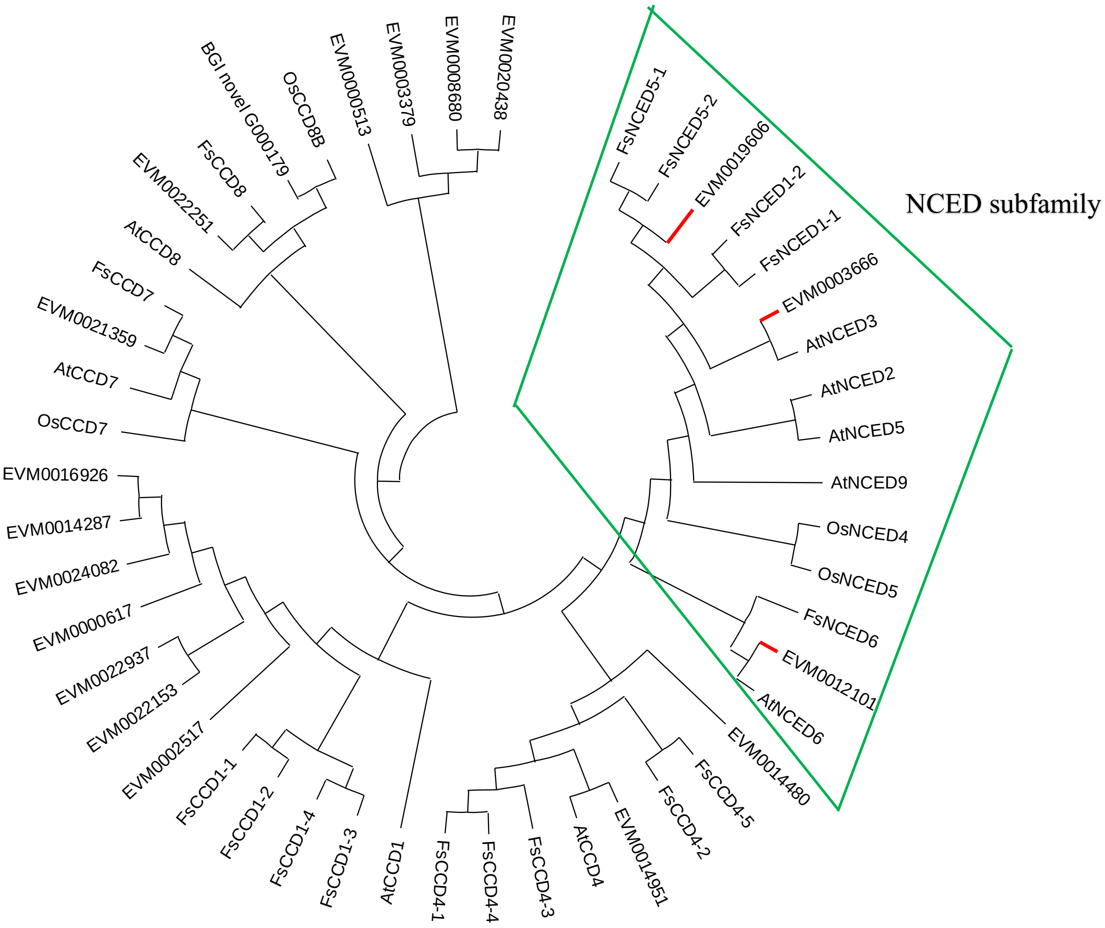

Figure S1. Phylogenetic analysis of *Xanthoceras*, Arabidopsis, rice, and *Forsythia suspensa* CCD genes.
