# Supplementary material for: Strigolactones Might Regulate Ovule Development after Fertilization in Xanthoceras sorbifolium
Source: Int J Mol Sci. 2024 Mar 14;25(6):3276. doi: 10.3390/ijms25063276 (PMC10969979; doi:10.3390/ijms25063276)
Supplement: Supplementary file 1 [file ijms-25-03276-s001.zip › Figure S2.pdf]

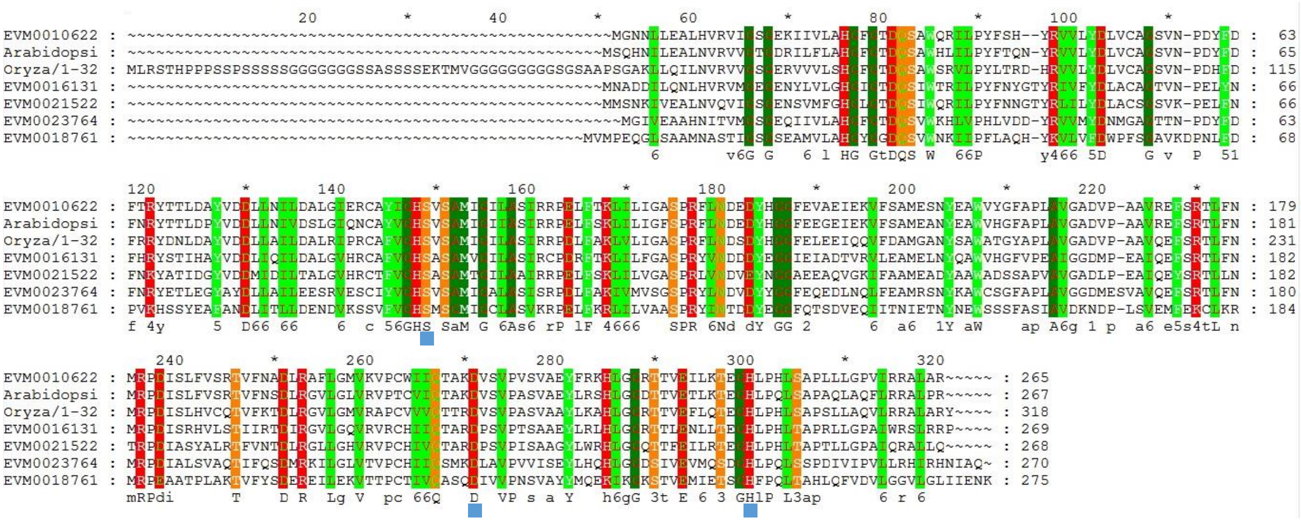

Figure S2. Sequence alignment and putative hydrolase catalytic triad of *Xanthoceras* D14 homolog proteins with rice D14 and Arabidopsis AtD14 proteins. The blue boxes indicate the conserved catalytic triad residues. Various color shading corresponds to the conservation of distinct amino acid residues.
