# Supplementary material for: Strigolactones Might Regulate Ovule Development after Fertilization in Xanthoceras sorbifolium
Source: Int J Mol Sci. 2024 Mar 14;25(6):3276. doi: 10.3390/ijms25063276 (PMC10969979; doi:10.3390/ijms25063276)
Supplement: Supplementary file 1 [file ijms-25-03276-s001.zip › Figure S4.pdf]

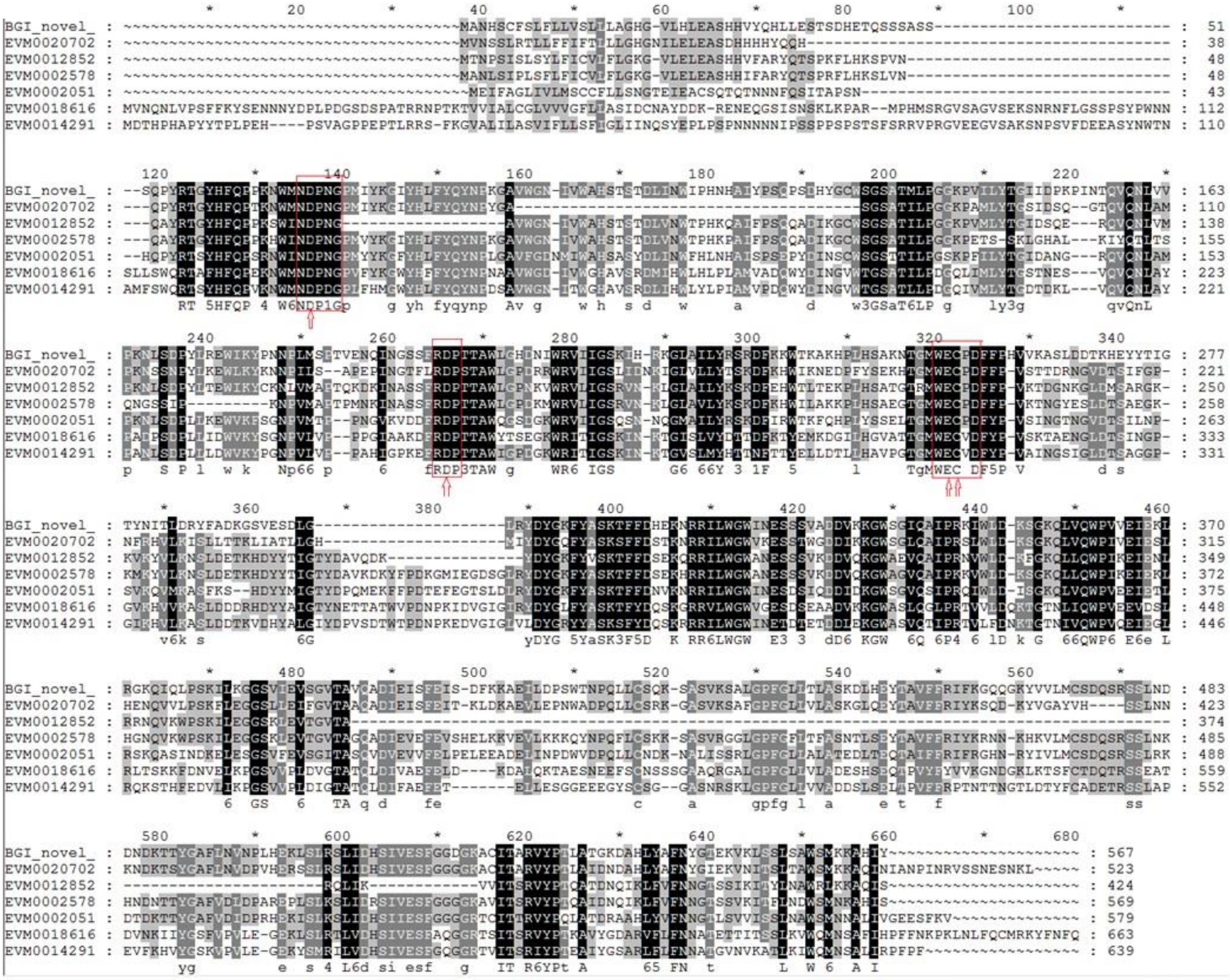

Figure S4. Alignment of the deduced protein sequences of the *Xanthoceras* CWINs and VINs. Three boxes show conserved sequence domains: NDPNG (β-fructosidase motif), RDP, and WECP(V)D, which correspond to the predicted active sites of the enzyme. Arrows show the catalytic residues. Various color shading corresponds to the conservation of distinct amino acid residues.
