# Supplementary material for: Strigolactones Might Regulate Ovule Development after Fertilization in Xanthoceras sorbifolium
Source: Int J Mol Sci. 2024 Mar 14;25(6):3276. doi: 10.3390/ijms25063276 (PMC10969979; doi:10.3390/ijms25063276)
Supplement: Supplementary file 1 [file ijms-25-03276-s001.zip › Figure S5.pdf]

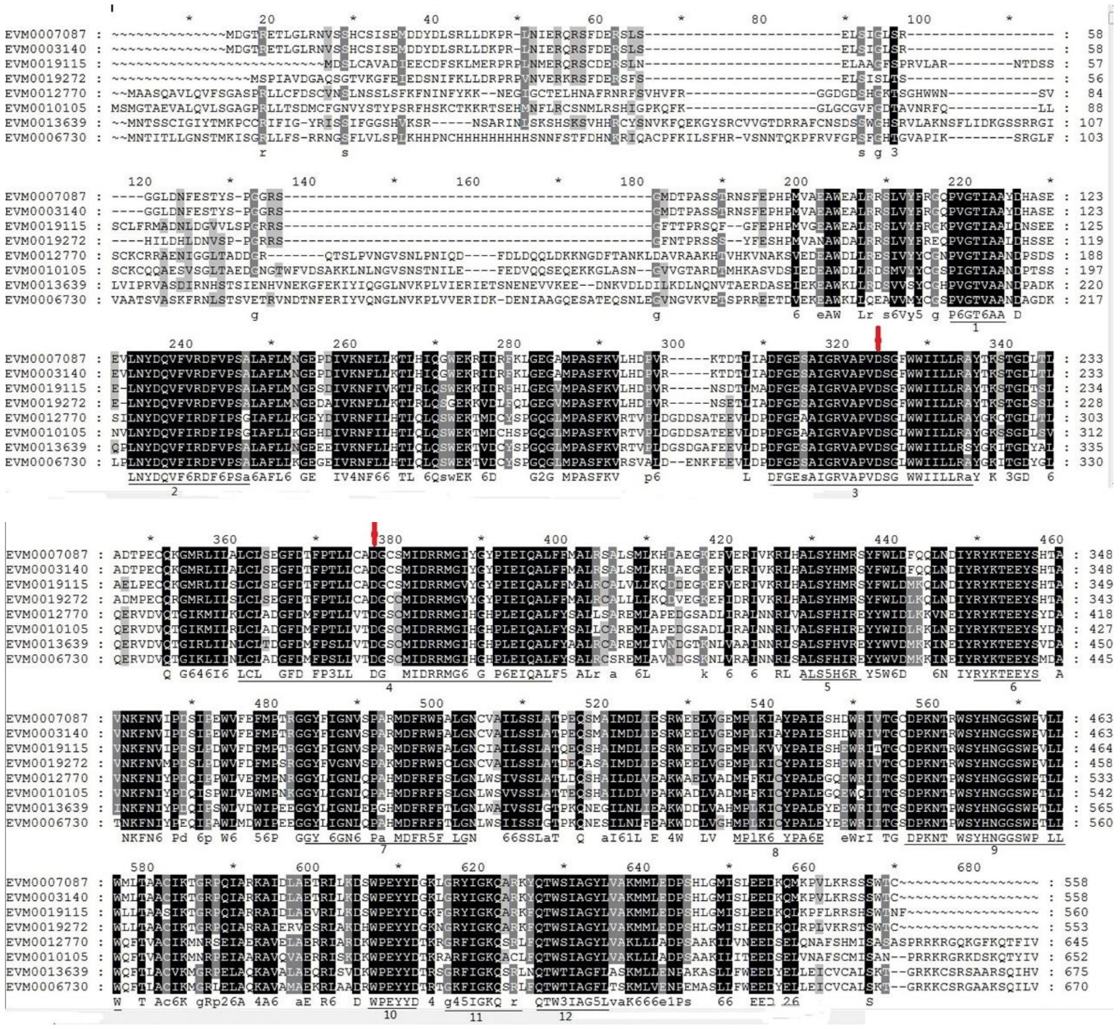

Figure S5. Alignment of the deduced protein sequences of the *Xanthoceras* alkaline/neutral invertases. Arrows show the amino acids most likely to correspond to active residues for neutral/alkaline invertases. 12 well-conserved regions were underlined. Various color shading corresponds to the conservation of distinct amino acid residues.
