# Supplementary material for: Strigolactones Might Regulate Ovule Development after Fertilization in Xanthoceras sorbifolium
Source: Int J Mol Sci. 2024 Mar 14;25(6):3276. doi: 10.3390/ijms25063276 (PMC10969979; doi:10.3390/ijms25063276)
Supplement: Supplementary file 1 [file ijms-25-03276-s001.zip › Table S4.pdf]

Table S4. Primers used in this study

Primer ID	Sequence(5'-3')	Gene Name
TRV2F	TTACTCAAGGAAGCACGATG	pTRV2
TRV2R	TCAATCAAGATCAGTCGAG	
TRV1F	CTTGAAGAAGAAGACTTTCGAAGTCTC	pTRV1
TRV1R	GTAAAATCATTGATAACAACACAGACAAAC	
Q6692	CCAGCGCTTCAGTCTCTTTC	*CWIN2*
Q6693	CATGGGTGCATTTGGATCGT	
Q6694	ATGTGTGGACTTCTACCCGG	*VIN1*
Q6695	GGTCATAGATCCCGAGTGCA	
Q6696	TGTGGGAGTGTGTGGACTTT	*VIN2*
Q6697	CATCCAGGCTTGCCTTAACC	
Q6698	CGAATCCAAGCTTGCCCTTT	*A/N-Inv1*
Q6699	TTTGATTGGAGCAACACCGG	
Q6700	GAATGTTCTCCGACGACCAC	*CCD7*
Q6701	ATGAACCTGACCTCTCCAGC	
Q6702	AGAAGGGGTCGATCGTCATC	*CCD8*
Q6703	GTCAGTAACAATGGGGTGCG	
Q6704	ACCCAAGTGTTCAAATCGCC	*NCED3-2*
Q6705	CGGCTCGAATAATGGGTTGG	
Q6706	TTGCTTGGGAACGAACATGG	*MAX1*
Q6707	TTAAGGTTATGGGGCAGCCA	
Q6708	AGCATGTTGACGAATCTGGC	*D27*
Q6709	ATCTGTTGCTATGCCTCCCA	
Q6710	ACTTGCCCCAACACTACCAT	*LBO*
Q6711	ATCCCCACTCTTCACAAGCA	
Q6712	CTTTACGCGCTACACCACTC	*D14*
Q6713	CCAGAATTCCGATCATGGCG	
Q6714	GTCCTCGGCCAAGTAAGAGT	*EVM0016131*
Q6715	ACCCTCGGTCAACAAGTTCT	
Q6718	CCAGTGTGAACTTCCCTCCT	*VPE2*
Q6719	TCTCCGATCCATCTTCTGCC	
Q6720	GCGATCAGTTGCCAACATCT	*VPE4*
Q6721	AAGAGAACTCCAAGGACCCG	
Q6722	CTTCCCTCACCGTCTACTCC	*MAX2*
Q6723	AGGAACGAAATCAGCTCCCA	
Q6724	GGCTGAGCTCACTTGTATGC	*EVM0002628*
Q6725	AAAACCGTTCCCACAAGCTC	
Q6726	ACTGTTCCGCCACATCTACA	*PHO2*
Q6727	TTTGGCTCCGTGATACCCTT	
Q6728	TGGACCTGACAAAGTTGGGA	*EVM0000315*

Q6729	CAGCATCATTCTTCCCACGG	
Q6730	ATAATCAGCAACGTCCGTGC	*EVM0002706*
Q6731	AGGGCTATTTTCAGGGTGCT	
Q6785	GTCAACCTCGGTGACCAACT	*ACO2*
Q6786	TCCCGGGTTGTAGAATGAAG	
Q6787	CAACTACCCTCCATGCCCTA	*ACO3*
Q6788	AGCTGCAGACCACTGACCTT	
Q6732	GTGGTCTTCTTCTCCTCCCC	Actin 2 (*EVM0010329*)
Q6733	TCCAAACAGTTCGGTCCAGT	
