# Supplementary figures and images for: Strigolactones Might Regulate Ovule Development after Fertilization in Xanthoceras sorbifolium
Source: Int J Mol Sci. 2024 Mar 14;25(6):3276. doi: 10.3390/ijms25063276 (PMC10969979; doi:10.3390/ijms25063276)

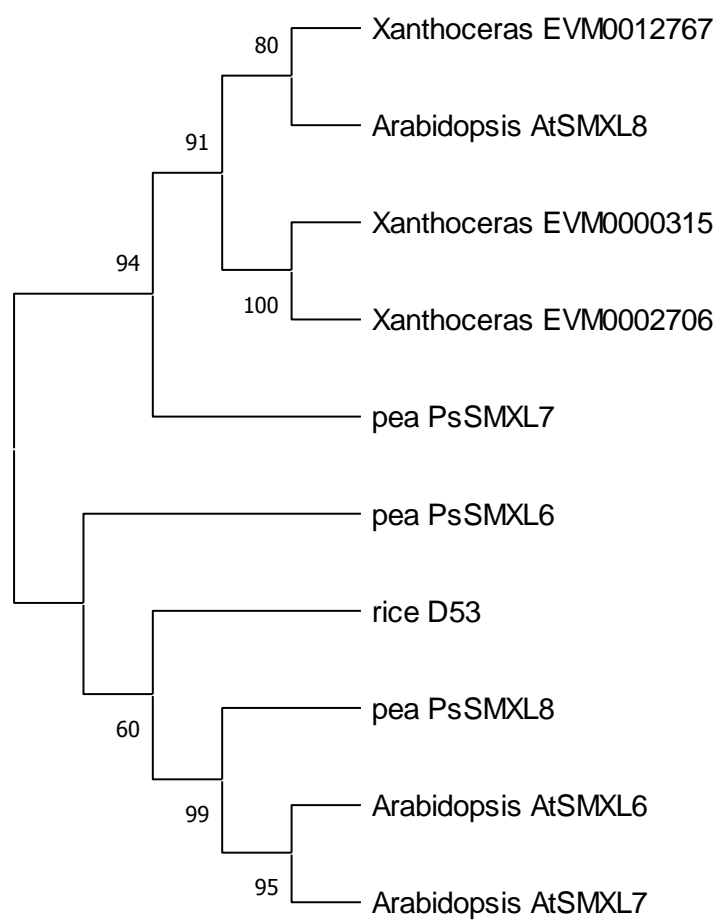

Figure S3. Phylogenetic analysis of SMXL homologs from *Xanthoceras*, *Arabidopsis*, and *rice*.

Supplement: Supplementary file 1 [file ijms-25-03276-s001.zip › Figure S3.pdf]
